# An advanced carbon/bismuth oxide nanoparticle-based filament for multifunctional 3D-printed electrochemical sensors toward paracetamol and lead in pharmaceutical, clinical, and environmental electroanalysis

**DOI:** 10.1007/s00604-026-08401-z

**Published:** 2026-09-14

**Authors:** Natália Marinho Caldas, Amanda Garcia Batista, Daniel M.-D. L. Alves, Samuel C. Silva, Diego A. Peixoto, Ariadne C. Catto, Valmor R. Mastelaro, Edson Nossol, Lucas V. de Faria, Diego P. Rocha, Bruno Campos Janegitz, Rafael Machado Dornellas

**Affiliations:** 1 Department of Analytical Chemistry, Institute of Chemistry, Fluminense Federal University, Niterói, 24020-141 RJ Brazil; 2 Institute of Chemistry, Uberlândia Federal University, Uberlândia, 38408- 100 MG Brazil; 3 Institute of Physics of São Carlos, University of São Paulo, São Carlos, 13566-590 SP Brazil; 4 Department of Analytical Chemistry, Institute of Chemistry, Federal University of Rio de Janeiro, Rio de Janeiro, 21941-909 Brazil; 5 Department of Chemistry, Federal Institute of Paraná, Pitanga, 85201-106 Paraná Brazil; 6 Department of Chemistry, Federal University of São Carlos, São Carlos, 13565-905 SP Brazil

**Keywords:** Additive manufacturing, Bismuth-based conductive filaments, Carbon composite materials, Analgesics analysis

## Abstract

3D printing offers a powerful, scalable approach to fabricating low-cost, high-performance electrochemical sensors for pharmaceutical and bioanalytical applications. We report the development of a 3D-printed graphite/polylactic acid (G/PLA) conductive composite electrode modified with bismuth oxide nanoparticles (Bi_2_O_3_NPs) to enhance paracetamol (PCT) detection. The optimized composition (G/Bi_2_O_3_NPs/PLA, 30:12.5:57.5% *wt.*) was processed by 3D printing to obtain electrodes without the need for complex post-treatment or electrochemical activation. Electrochemical impedance spectroscopy (EIS) revealed a marked decrease in the charge-transfer resistance (Rct) after Bi_2_O_3_NPs incorporation, indicating improved interfacial electron transfer. Electrochemical characterization demonstrated a significant increase in the PCT oxidation current and a shift in the peak potential toward less positive values, indicating the electrocatalytic role of Bi_2_O_3_NPs. Under optimized batch injection analysis with amperometric detection (BIA-AD) conditions, the sensor exhibited a wide linear dynamic range of 0.05–300 µmol L⁻¹, a low limit of detection of 0.006 µmol L⁻¹, and precision (RSD < 5%). Inter-electrode reproducibility was satisfactory (RSD < 5%), highlighting the robustness of the fabrication process. The proposed platform also demonstrated applicability to Pb(II) detection, evidencing the versatility of the G/Bi_2_O_3_NPs/PLA composite and expanding its potential for environmental monitoring and heavy metal analysis. Furthermore, selectivity studies conducted in the presence of common interferents showed negligible changes in the PCT response, confirming the sensor’s reliability for pharmaceutical quality control applications. Overall, the 3D-printed G/Bi_2_O_3_NPs/PLA electrode combines analytical performance with simplicity of fabrication, low cost, and scalability, positioning it as a promising alternative to conventional electrochemical sensors for routine PCT determination.

**Graphical Abstract:**

**Figure:**
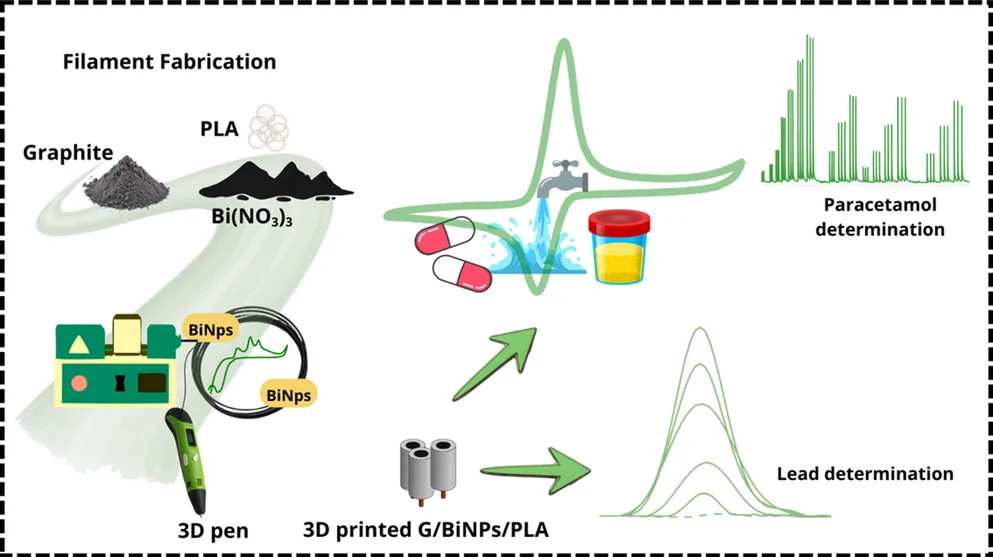

**Supplementary Information:**

The online version contains supplementary material available at https://doi.org/10.1007/s00604-026-08401-z.

## Introduction

For many decades, bismuth has been employed in the development of electrochemical transducers for a wide range of applications [1]. It has been extensively used in the fabrication of thin films as an environmentally friendly alternative to mercury for the determination of metals at cathodic potentials [1, 2]. However, other applications can also be found in the literature, particularly for the determination of organic compounds, owing to its electrochemical properties, high sensitivity, and its ability to maintain the long-term stability of sensors by preventing oxidation-driven degradation [3–5]. Additional advantageous features of bismuth for the development of electrochemical transducers include its good catalytic activity, environmental friendliness, and cost-effectiveness [1, 2].

Despite these favorable characteristics, bismuth remains largely underexplored in the development of sensors manufactured by 3D printing technologies [6–8]. It is worth noting that additive manufacturing, particularly fused filament fabrication (FFF), has proven to be a versatile, environmentally sustainable, and low-cost approach for the on-demand fabrication of electroanalytical devices [9, 10]. In this context, Mertiri and colleagues proposed the digital fabrication of 3D-printed thermoplastic sensors in situ modified with “green” spark-discharge-generated bismuth particles [8]. Although promising results were achieved, this strategy requires additional processing steps during sensor fabrication, which may lead to reproducibility issues and increased manufacturing costs. Conversely, Okpara et al. developed a polylactic acid (PLA)–based sensor composed of carbon black, castor oil, and Bi₂O₃ [7], while Tsetoni et al. proposed a bismuth- and carbon-based filament produced via a solvent-casting method [6]. In both studies, the authors engineered custom filaments containing the aforementioned components for electrochemical applications. Their sensors exhibited satisfactory performance for the stripping determination of lead and, to the best of our knowledge, are among the few studies employing bismuth-based compounds in the production of 3D-printing filaments, underscoring that this field still requires further exploration for broader analytical applications.

Bismuth-based sensors can be found in the literature for the enhanced determination of paracetamol (PCT) [11–13], which is one of the most widely used drugs in the world, acting as an antipyretic, analgesic, and non-steroidal anti-inflammatory compound [14, 15]. When correctly administered, it does not pose health risks; however, indiscriminate use can cause severe liver damage [11]. Additionally, PCT is considered an emerging contaminant, as its misuse or improper disposal can result in contamination of aqueous environments [16]. Therefore, monitoring this compound in different matrices is of paramount importance.

In this context, the present work reports the development of a graphite- and bismuth-based filament dispersed in a PLA thermoplastic matrix, aimed at enabling the fabrication of enhanced, low-cost, and environmentally friendly sensors with design freedom. As a proof of concept, the sensitive and selective determination of PCT in water, tablet, and synthetic urine samples is presented. Furthermore, the proposed platform was successfully applied to the electrochemical sensing of Pb(II), a toxic heavy metal of environmental concern [17]. The results highlight the versatility of the proposed material and its potential for electrochemical sensing of both organic and inorganic analytes, broadening the applicability of 3D-printed platforms for diverse analytical purposes.

## Experimental

### Reagents and solutions

Deionized water with a conductivity of 0.055 µS cm⁻¹ was acquired from a Sartorius Arium^®^ Pro purification system (Göttingen, Germany). Graphite (G) powder (particle size < 20 μm, 98.0% *wt.*), galactose monohydrate (GAL, 99.0% *wt.*), glucose (GLU, 99.0% *wt.*), and urea (U, 99% *wt.*) were purchased from Sigma-Aldrich (St. Louis, USA). Bismuth(III) nitrate (Bi(NO_3_)_3_, 80.0% *wt.*) and Starch (STC, 99.6% *wt.*) were purchased from ISOFAR (Rio de Janeiro, Brazil). Potassium chloride (KCl, 99.5% *wt.*) and potassium hydroxide (KOH, 85% *wt.*) were obtained from Merck (Darmstadt, Germany). Paracetamol (PCT, 99.0% *wt.*) was purchased from Formulando (Rio de Janeiro, Brazil). Sodium lauryl sulfate (SDS, 90.0% *wt.*) and potassium ferrocyanide ([Fe(CN)_6_]^4−^, 99.0% *wt.*) were supplied by Vetec (Rio de Janeiro, Brazil). Hydrochloric acid (HCl, 37.0% v/v) was obtained from J.T. Baker (Goiânia, Brazil). Chloroform (99.5% v/v) was purchased from Synth (São Paulo, Brazil), as well as acetic acid (99.5% v/v), phosphoric acid (85.0% v/v), and boric acid (98.0% *wt.*) from Synth (Diadema, Brazil). Lead stock solution (Pb(II), 1000 mg L^−1^) was purchased from Haloquimica Ltda (São Paulo, Brazil). PLA and acrylonitrile butadiene styrene (ABS) pellets and filament were obtained from GTMax 3D (São Paulo, Brazil).

### Sample acquisition and preparation

In this work, three distinct sample matrices were investigated for PCT determination, including tap water, pharmaceutical tablets, and synthetic urine. The tap water sample was directly collected from the water supply of our research laboratory. Pharmaceutical tablets containing PCT were acquired from local drugstores in Niterói, Rio de Janeiro, Brazil. According to the manufacturers, each tablet contained 750 mg of PCT. The synthetic urine sample was prepared following the procedure reported by Laube et al. [18]. The formulation consisted of an aqueous mixture containing 7.48 mmol L⁻¹ CaCl₂·2 H₂O, 49.6 mmol L⁻¹ NaCl, 15.5 mmol L⁻¹ Na₂SO₄, 10.3 mmol L⁻¹ KH₂PO₄, 21.5 mmol L⁻¹ KCl, 18.7 mmol L⁻¹ NH₄Cl, and 0.42 mol L⁻¹ urea. After preparation, the pH of the urine sample was adjusted to 6.0.

A simple and practical procedure was employed to prepare the pharmaceutical samples. Initially, 0.78 g of the tablet sample was accurately weighed, transferred to a 100 mL volumetric flask, and diluted to the mark with 0.12 mol L⁻¹ BR buffer (pH 6.0). The resulting solution was then subjected to simple filtration. Subsequently, a 0.52 mL aliquot of the filtrate was transferred to a 25 mL volumetric flask and diluted to the mark with 0.12 mol L⁻¹ BR buffer (pH 6.0). Finally, the prepared solution was injected directly into the BIA electrochemical cell. In contrast, the tap water and synthetic urine samples were subjected to a 10-fold dilution. The samples were spiked with PCT at three concentration levels (20,40 and 60 µmol L⁻¹) to evaluate the accuracy of the proposed method.

### Instrumentation

Fourier transform infrared (FTIR) spectra were obtained using a PerkinElmer Frontier MIR/FIR spectrophotometer (USA) in attenuated total reflectance (ATR) mode, with an accessory purchased from Pike Technologies (United States). Raman spectroscopy was performed using an Ar ion laser at 532 nm with an incident power of 2%. The spectra were recorded using a LabRAM HR Evolution Raman microscope (HORIBA, Japan).

Scanning electron microscopy (SEM) images were acquired at 20 kV using a Tescan Vega 3 microscope (Czech Republic) with a secondary electron detector. Energy-dispersive X-ray spectra (EDS) were obtained from SEM images acquired with the appropriate INCA X-Act detector from Oxford Instruments (UK).

An analysis of the sample surface was performed using X-ray photoelectron spectroscopy (XPS) on a Scienta Omicron ESCA spectrometer (Scienta Omicron, Uppsala, Sweden) with Al Kα monochromatic radiation (1486.7 eV). The obtained data were adjusted using CasaXPS software and calibrated using the adventitious carbon binding energy (284.8 eV).

A conventional three-electrode system was employed for all electrochemical measurements. The working electrode was a 3D-printed G/Bi_2_O_3_NPs/PLA electrode, a saturated Ag|AgCl|KCl electrode served as the reference electrode, and pencil graphite served as the counter electrode. All electrodes were interfaced with a potentiostat/galvanostat, Ivium CompactStat Technologies^®^ (Eindhoven, The Netherlands, model B09118), operated via IviumSoft^®^ software, which controlled and recorded electrochemical techniques, including cyclic voltammetry (CV), square-wave voltammetry (SWV), electrochemical impedance spectroscopy (EIS), and chronoamperometry. For the BIA experiments, a custom 3D-printed electrochemical cell, was used for the electrochemical sensors developed in this work; it was fabricated from ABS filament, following a design previously reported elsewhere by our research group [19]. The working electrode was positioned at the bottom of the cell in a wall-jet configuration [20], approximately 2 mm from the dispensing tip of an Eppendorf Multipette^®^ E3 electronic micropipette (Hamburg, Germany). This system allows programmable injections ranging from 10 to 1000 µL at dispensing rates between 16.9 and 298.5 µL s⁻¹. The reference and counter electrodes were placed in the upper portion of the electrochemical cell.

To validate the electrochemical results for PCT detection, the samples were also examined using the official UV–Vis method. The analyses were performed on an Agilent Cary 60 UV–Vis spectrophotometer (California, USA) operating in scanning mode from 200 to 800 nm with a 1 cm quartz cuvette. The absorbance used for quantification was measured at 245 nm.

### Preparation of G/Bi_2_O_3_NPs/PLA filament and fabrication of 3D-printed electrochemical sensors

BiNP-modified filaments were produced by mixing G, PLA, and Bi(NO₃)₃ at defined mass ratios. Four compositions were prepared: G/PLA (30:70% *wt.*) and G/Bi_2_O_3_NPs/PLA with 7.5, 10, and 12.5% *wt.* of Bi(NO₃)₃ and 62.5, 60, and 57.5% *wt.* of PLA. The G content was fixed at 30% *wt.* to ensure adequate electrochemical response, while PLA (≥ 57.5% *wt.*) provided mechanical stability.

PLA pellets were dissolved in ~ 150 mL of acetone/chloroform (3:1 v/v) at 70 °C under stirring. Bi(NO₃)₃ and G were then gradually added, and the mixture was stirred for 1 h. The suspension was cast onto parchment paper, dried at 25 °C for 24 h, cut, and extruded using a FILMAQ^®^ extruder (Curitiba, Brazil) at 195 °C and at maximum speed, yielding filaments with a diameter of 1.75 mm. Materials were stored in sealed containers at room temperature.

The ABS-based electrode body was designed in SolidWorks^®^ and printed using a Creality K1 (Shenzhen, China), with dimensions of 4 cm in length, 0.75 cm in outer diameter, and 0.50 cm in inner diameter. Printing conditions were: 0.4 mm nozzle, 230 °C extrusion temperature, 100 °C bed temperature, 0.3 mm layer height, 100 mm s⁻¹ print speed, three walls, and 100% infill.

An internal cavity was included to accommodate the insertion of a copper wire and ensure electrical contact. The conductive filament was deposited over an area of 0.196 cm^2^ using a Sanmersen 3D pen (Shenzhen, China) at 190 °C. Before measurements, the surface was polished with 600 and 1200-grit sandpapers.

## Results and discussion

### Characterization of composite materials

**Fig. 1:**
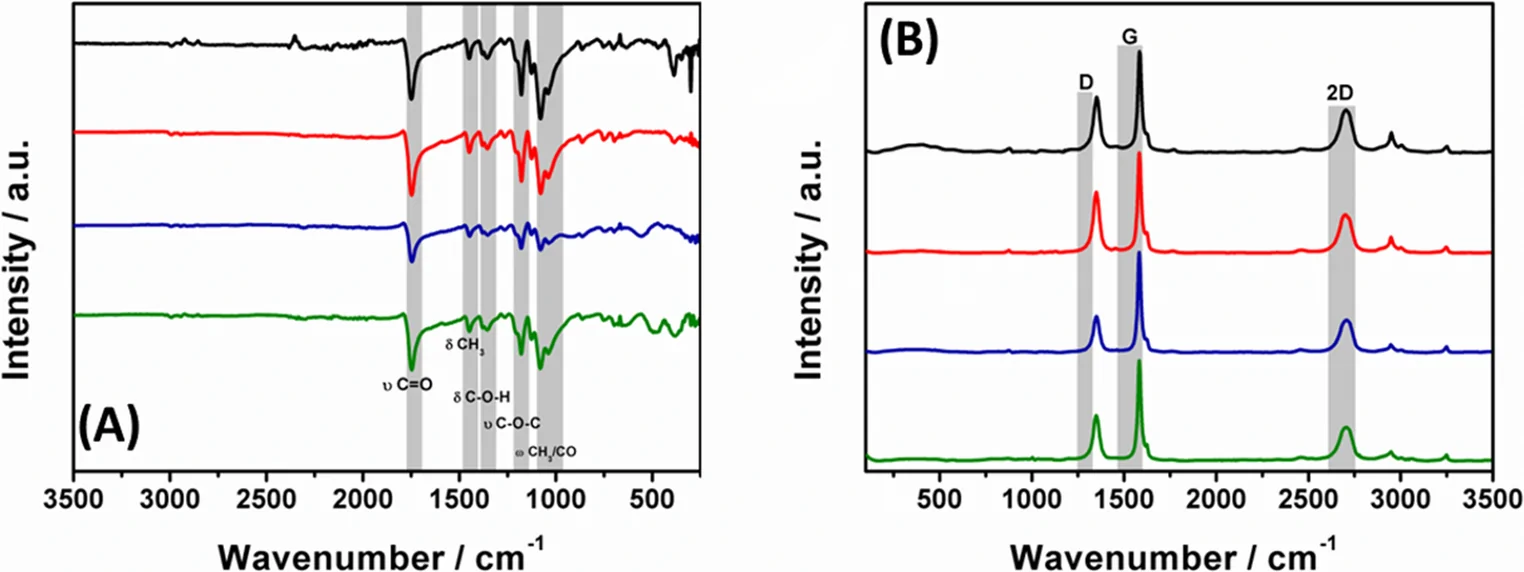
(**A**) FTIR-ATR and (**B**) Raman spectra obtained from G/PLA (30:70% *wt.*, black line), G/Bi_2_O_3_NPs/PLA (30:7.5:62.5% *wt.*, red line), G/Bi_2_O_3_NPs/PLA (30:10:60% *wt.*, blue line), and G/Bi_2_O_3_NPs/PLA (30:12.5:57.5% *wt.*, olive line) electrodes

The FTIR spectra of the prepared electrodes are presented in Fig. 1A. The presence of PLA was confirmed by characteristic vibrational modes of oxygenated groups, including C = O stretching at 1743 cm⁻¹, C-O-H stretching at 1347 cm⁻¹, and C-O-C stretching at 1177 cm⁻¹, as well as overlapping CH₃/CO rocking vibrations and C-O-C stretching between 1085 and 1035 cm⁻¹. Additionally, a band at 1451 cm⁻¹ was observed, corresponding to the CH₃ bending mode [21, 22]. The inherently low dipole moment of the C = C bond, which predominantly constitutes graphite-like structures, accounts for the weak, and in most cases absent, vibrational modes observed in the FTIR-ATR spectrum. The superposition, shift, and broadening of the vibrational bands in the carbonaceous phase are attributed to direct interactions between oxygenated and non-oxygenated functional groups in PLA. Consequently, these interactions hinder a clear structural distinction between the PLA/G support matrices when analyzed via FTIR spectroscopy. No bands attributable to the bismuth precursor salt or any compound formed during the electrode surface modification process were detected.

The Raman spectra of the analyzed electrodes are presented in Fig. 1B, in which the characteristic graphite peaks are more intense than those of PLA. This difference arises from the high polarizability of π electrons in graphite C = C bonds, which leads to more intense Raman scattering. The G band at 1585 cm⁻¹ corresponds to in-plane vibrations of sp² carbon atoms in a trigonal arrangement, while the D band at 1351 cm⁻¹ is associated with the vibrational mode of six-membered sp² carbon rings, indicating defects in the graphitic structure caused by heteroatoms or sp³ carbons. The 2D band between 2500 and 3000 cm⁻¹ arises from a second-order scattering process associated with an overtone of the D band and is characteristic of the ordered stacking of graphene sheets in the graphite structure. These results demonstrate the distinctive structural features of the investigated electrodes [23, 24]. Similar to the FTIR spectra, no peaks related to bismuth species were observed.

The structural defects in the carbon structures of the studied electrodes were quantified using the I_D_/I_G_ ratio, which represents the intensity, or, preferably, in this case, the integrated area, of the D band and G band, respectively. The D band, typically located near 1350 cm^−1^, is associated with defect-activated vibrational modes in graphitic carbon, including structural imperfections, edge sites, and finite crystallite dimensions. In contrast, the G band, generally observed near 1580 cm^−1^, corresponds to the in-plane stretching vibration of sp²-hybridized carbon atoms. Thus, I_D_/I_G_ is a dimensionless Raman parameter commonly used to estimate the relative degree of structural disorder in graphitic and other carbonaceous materials. For carbon materials within the low-to-moderate disorder regime, an increase in I_D_/I_G_ generally indicates a greater concentration of defects, a higher proportion of edge sites, and/or a reduction in the size of ordered sp²-carbon domains [25–27].

The calculated I_D_/I_G_ values for the different electrodes are presented in Table S1. The results suggest that, for Bi contents up to 7.5% *wt.* in the material, defect density increases. At higher Bi percentages in the electrode composition, the number of defects decreased, indicating that decorating graphite with Bi nanoparticles may fill sheet defects, yielding a less defective material. Accordingly, the increase in I_D_/I_G_ observed for bismuth contents up to 7.5% *wt.* suggests that bismuth incorporation promotes additional disorder or disrupts the local graphitic structure of the electrode material. At higher Bi contents, the decrease in I_D_/I_G_ may indicate a reduction in the Raman-active defect population, partial restoration of graphitic ordering, changes in the carbon microstructure, or the formation of Bi-rich regions that modify the carbon Raman response.

**Fig. 2:**
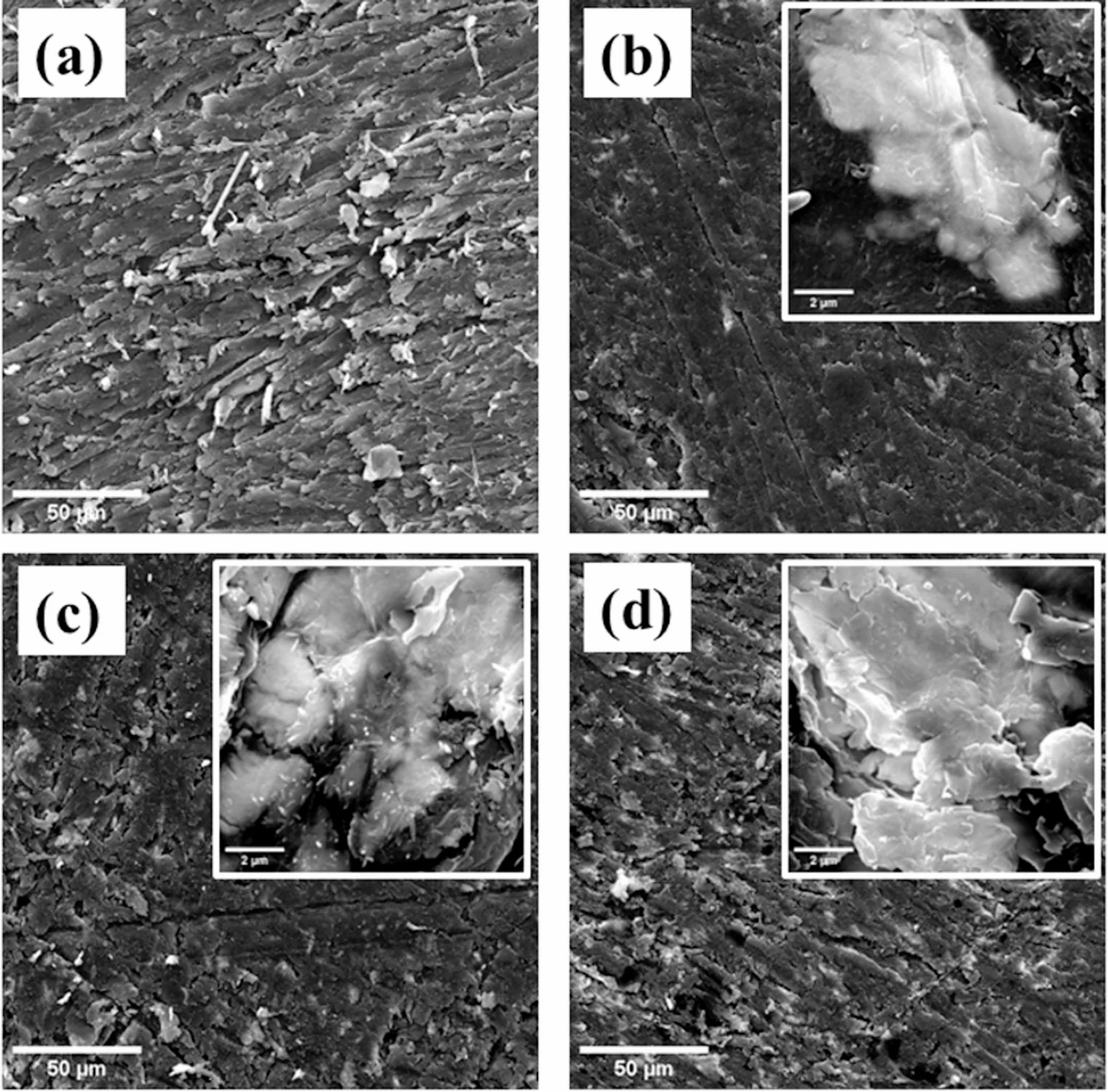
SEM images of (**a**) unmodified G/PLA (30:70% *wt.*), (**b**) G/Bi2O3NPs/PLA (30:7.5:62.5% *wt.*), (**c**) G/Bi2O3NPs/PLA (30:10:60% *wt.*), and G/Bi2O3NPs/PLA (30:12.5:57.5% *wt.*) electrodes

The SEM images acquired at the same magnification revealed a homogeneous distribution of the carbonaceous material and polymer within the PLA/G matrix, with a uniform surface morphology characterized by rough features, as shown in Fig. 2a. In the material with added Bi salt, distinct surface spots were observed (Fig. 2b-d), attributed to the formation of Bi_2_O_3_NPs. Morphological details became more evident in the 20k×-magnified images, where Bi nanoparticles were clearly visible. This morphological characterization confirmed that the electrochemical modification process effectively incorporated Bi_2_O_3_NPs into the G/PLA matrix.

The EDS measurements of the electrode surfaces (Figure S1A) confirmed the expected elemental composition and verified the successful incorporation of Bi. The spectra showed an intense gold peak from the conductive coating required for analysis, along with carbon and oxygen signals corresponding to the G/PLA composite matrix. Most significantly, the appearance of characteristic Bi peaks in the spectra of modified electrodes (Figure S1-b-d) provided conclusive evidence of successful modification, demonstrating effective integration of Bi_2_O_3_NPs into the PLA/G matrix. The EDS data complement the morphological observations, providing a comprehensive view of the electrode modification process.

**Fig. 3:**
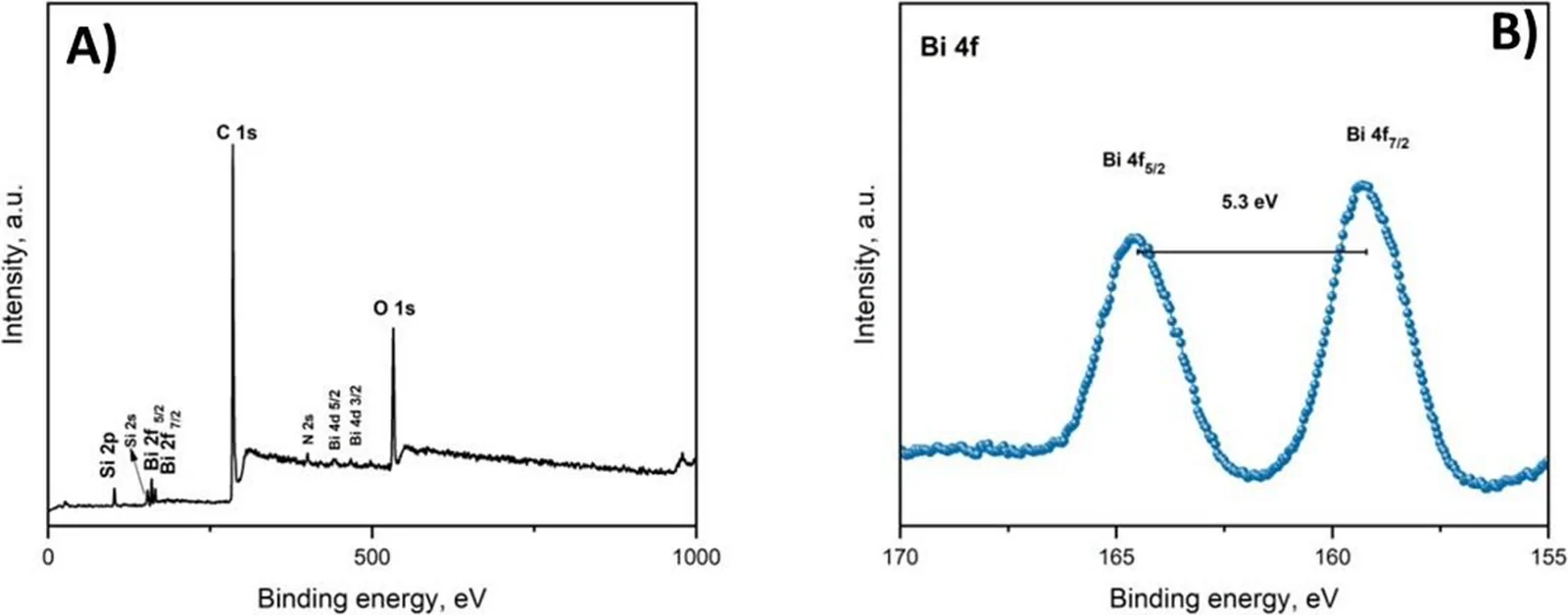
(**A**) Survey spectrum of the electrode containing 12.5% *wt.* Bi_2_O_3_NPs. (**B**) High-resolution Bi 4f spectrum

XPS was employed to characterize the surface chemical states and elemental composition of the electrode. Figure 3A shows the survey spectrum of the sample, revealing characteristic peaks corresponding to Bi, N, Si, O, and C. Figure 3B presents the high-resolution Bi 4f XPS spectrum. The spectrum exhibits two main peaks at 159.2 eV and 164.5 eV, corresponding to the Bi 4f₇/₂ and Bi 4f₅/₂ components, respectively. These binding energy values, along with the 5.3 eV spin–orbit splitting, are consistent with those reported for Bi₂O₃, indicating the presence of Bi³⁺ species [28–30].

The C 1s spectrum of the sample (Figure S2A) was deconvolved into five components corresponding to the bonds in graphitic (sp^2^) carbon, C-C(sp^3^), C-O, C = O, and O-C = O [31–33]. The O 1s spectrum of the sample shows a broad and asymmetric profile, which can be deconvoluted into three components located at 530.1 eV, 531.7 eV, and 533.2 eV (Figure S2B). The lowest-binding-energy component is attributed to lattice oxygen associated with Bi–O bonds, while the higher-binding-energy components are associated with oxygen species bonded to carbon, such as C–O and C = O groups present on the sample surface [34–36].

### Electrochemical characterization of 3D-printed G/Bi_2_O_3_NPs/PLA sensors

The electrodes were electrochemically evaluated to investigate the Bi oxidation and reduction processes. For this, the electrochemical behavior of the following compositions: G/PLA (30:70% *wt.*, black line), G/Bi_2_O_3_NPs/PLA (30:7.5:62.5% *wt.*, red line), G/Bi_2_O_3_NPs/PLA (30:10:60% *wt.*, blue line), and G/Bi_2_O_3_NPs/PLA (30:12.5:57.5% *wt.*, olive line) was investigated in the presence of 0.1 mol L⁻¹ KCl (supporting electrolyte). As illustrated in Fig. S3A, oxidation processes characteristic of Bi species were observed in the potential range between − 0.6 and + 0.3 V vs. Ag|AgCl|KCl_(sat.)_ for surfaces containing Bi_2_O_3_NPs. Furthermore, a progressive increase in the intensity of these signals was observed with increasing Bi_2_O_3_NPs content, indicating the successful incorporation of these nanoparticles. The anodic region of the voltammogram exhibits three well-defined oxidation peaks, as reported for electrodeposited Bi thin films [37, 38]. The observed peaks are attributed to electrochemical processes involving (Bi₂O₃NPs), which may lead to the formation and/or transformation of trivalent bismuth species (Bi³⁺) and different bismuth oxide or hydroxide phases. Although these processes occur at distinct potentials, they converge to the same oxidation state, as evidenced by a single cathodic reduction peak in the reverse scan. This behavior is entirely consistent with the results obtained through SEM, EDS, FTIR-ATR, and XPS analyses.

Later, the electrodes were evaluated using the ferricyanide/ferrocyanide redox couple [Fe(CN)_6_]^3−/4−^ to assess their electrochemical behavior. For this, additional cyclic voltamograms were recorded at a scan rate of 50 mV s^−1^ (Figure S4A). Also, EIS measurements were recorded (Figure S4B). As depicted, the electrodes containing 12.5% *wt.* Bi_2_O_3_NPs (olive green line) exhibit a notably superior electrochemical response compared to the non-modified electrode (black line), as evidenced by increased anodic and cathodic peak currents and a marked reduction in the semicircle diameter in the Nyquist diagrams. The electrode containing 12.5% *wt.* exhibited the lowest charge-transfer resistance (1.6 kΩ), compared with the control electrode (12.54 kΩ). Furthermore, a curious behavior is observed: the addition of Bi_2_O_3_NPs leads to a progressive decrease in the semicircle diameter and a simultaneous increase in anodic and cathodic peak currents, indicating reduced charge-transfer resistance, enhanced electroactive surface area, and improved electron-transfer kinetics at the electrode interface. The quality of the fitting was further assessed by the chi-square (χ²) values obtained from the equivalent circuit analysis. The G/PLA (30:70% *wt.*), G/Bi₂O₃NPs/PLA (30:7.5:62.5% *wt.*), G/Bi₂O₃NPs/PLA (30:10:60% *wt.*), and G/Bi₂O₃NPs/PLA (30:12.5:57.5% *wt.*) electrodes exhibited χ² values of 1.43 × 10^−4^, 4.34 × 10^−4^, 3.08 × 10^−4^, and 2.96 × 10^−4^, respectively. These low χ² values indicate good agreement between the experimental impedance spectra and the fitted data, confirming that the adopted Randles equivalent circuit provides an appropriate representation of the electrochemical behavior of the fabricated electrodes.

Next, studies varying the scan rate were performed for all electrodes using the redox pair [Fe(CN)₆]^3−/4−^. This study was employed to determine the standard heterogeneous electron transfer rate constant (k^0^), applying the Nicholson method [39]. Cyclic voltammograms were recorded at scan rates of 10–100 mV s^−1^ for the non-modified and Bi_2_O_3_NPs-based electrodes, as shown in Figure S5. The electrode modified with 12.5% *w.* of Bi_2_O_3_NPs exhibited a markedly enhanced kinetic response (k⁰ = 2.1 × 10^−3^ cm s^−1^) compared to the Bi_2_O_3_NPs-free surface (k⁰ = 1.8 × 10^−4^ cm s^−1^), indicating that the electron transfer process occurs approximately 12 times faster at the Bi_2_O_3_NPs-incorporated electrode.

For the [Fe(CN)₆]^3−/4−^ redox probe, the electrochemical response is consistent with a reversible single-electron transfer process. The corresponding Randles–Ševčík plots (insets in Figure S5(A-D)) show a linear dependence of the peak current on the square root of the scan rate, confirming that the redox reactions are governed by diffusion-controlled kinetics [36]. Based on the Randles–Ševčík approach, the electroactive surface areas were estimated as 1.34, 1.71, 3.48, and 4.82 mm² for the G/PLA (30:70% *wt.*), G/Bi_2_O_3_NPs/PLA (30:7.5:62.5% *wt.*), G/Bi_2_O_3_NPs/PLA (30:10:60% *wt.*), and G/Bi_2_O_3_NPs/PLA (30:12.5:57.5% *wt.*) electrodes, respectively. Notably, the electrode containing 12.5% % *wt.* Bi_2_O_3_NPs exhibited the largest electroactive area. The main electrochemical parameters, including the cathodic peak current (*I*_*pca*_), peak separation (*ΔE*_*p*_), standard heterogeneous electron transfer rate constant (k⁰), and electroactive surface area (Aₑ), are summarized in Table S2.

### Determination of PCT

**Fig. 4:**
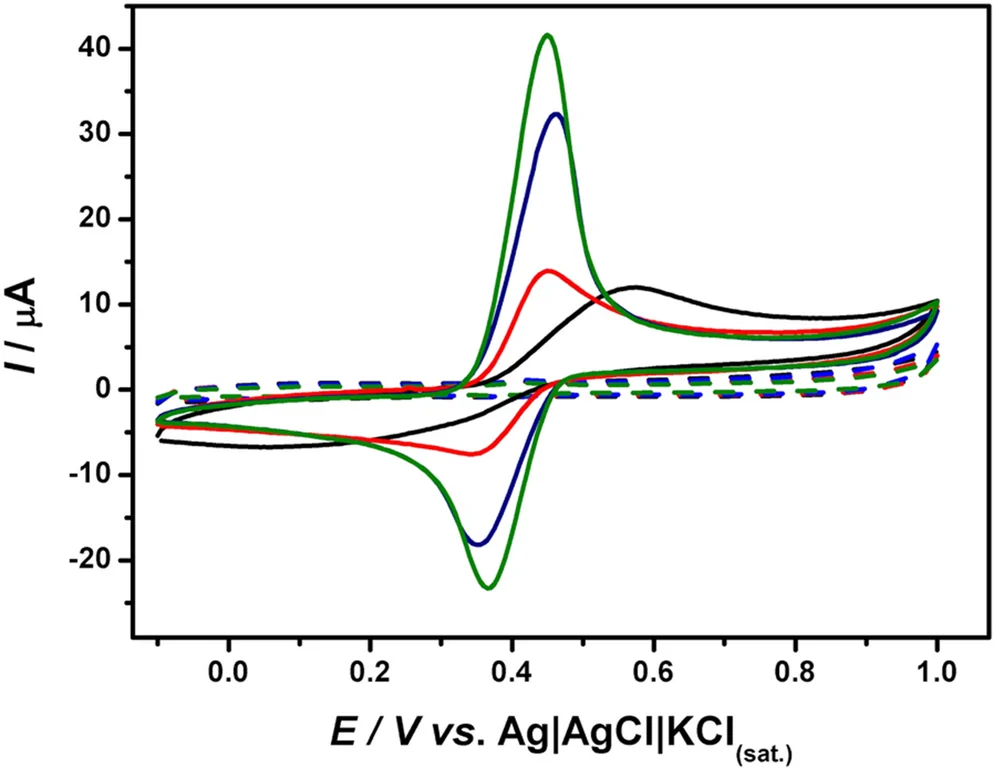
CVs recorded in the presence of 0.3 mmol L^−1^ PCT in 0.1 mol L^−1^ BR buffer (pH 6.0) using the G/PLA (30:70% *wt.*, black line), G/Bi_2_O_3_NPs/PLA (30:7.5:62.5% *wt.*, red line), G/Bi_2_O_3_NPs/PLA 30:10:60% *wt.*, blue line), and G/Bi_2_O_3_NPs/PLA (30:12.5:57.5% *wt.*, olive line). The dashed lines correspond to the respective blanks. Experimental conditions: recorded at a scan rate of 50 mV s^−1^ within the potential range of − 0.1 to 1.0 V and a step potential of 5 mV

Next, the same surfaces were examined for the electrochemical oxidation of 0.3 mmol L^−1^ PCT in 0.1 mmol L^−1^ BR buffer, pH 6.0 (see Fig. 4). PCT was chosen as a proof of concept because it is a molecule widely analyzed in Bi-modified sensors, in order to demonstrate the enhanced performance of the new material developed via 3D printing. Different electrochemical responses were observed, showing a progressive improvement in PCT oxidation performance with increasing Bi_2_O_3_NPs content, culminating in the most significant *I*_*p*_ and *E*_*p*_ values for the electrode containing 12.5% *wt.* Bi_2_O_3_NPs. Table S3 summarizes the results obtained from Fig. 4. It should be noted that the unmodified electrode (G/PLA (30:70% *wt.*, black line)) exhibited the least efficient performance, with a less clearly defined electrochemical profile. Incorporating Bi_2_O_3_NPs improved electrocatalytic performance, as shown by favorable shifts in the oxidation peak potential, an increased heterogeneous electron-transfer rate constant (k⁰), and reduced charge-transfer resistance (Rct), along with increased electroactive surface area. Given this and the enhanced electrochemical characteristics observed for this composition, the G/Bi_2_O_3_NPs/PLA electrode (30:12.5:57.5% *wt*., olive-green line) was selected for subsequent experiments.

**Fig. 5:**
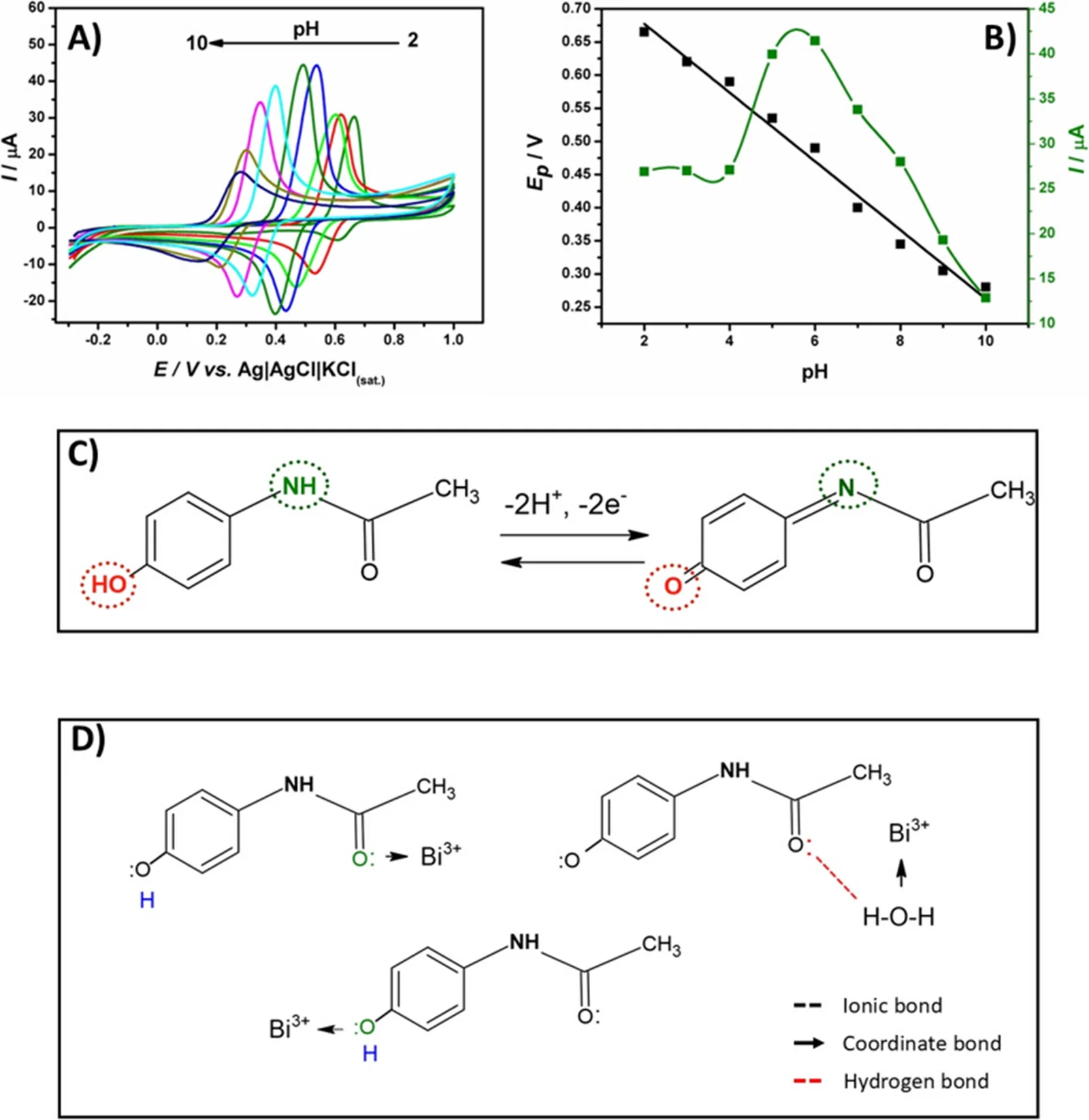
**(A) **CVs recorded in the presence of 0.3 mmol L^−1^ PCT upon the 3D-printed G/Bi_2_O_3_NPs/PLA (30:12.5:57.5% *wt.*) electrode at different pH values (2–10) using 0.12 mol L^−1^ BR buffer as background electrolyte recorded at a scan rate of 50 mV s⁻**¹** within the potential range of − 0.2 to 1.0 V. The current and potential axes are expressed in µA and V, respectively. The voltammograms illustrate the effect of pH on the electrochemical oxidation response of PCT. (**B**) Relationship between pH and *E*_p_ (black squares) and between pH and *I*_p_ (olive squares). (**C**) Proposed mechanism for the electrochemical oxidation of PCT. (**D**) Proposed possible interactions between PCT and Bi_2_O_3_NPs

The effect of the pH of the supporting electrolyte was systematically investigated in Bi_2_O_3_NPs-modified 3D-printed sensor (12.5% *wt.*) in a pH range of 2 to 10, using 0.12 mol L^− 1^ BR buffer. As illustrated in Fig. 5A, the oxidation and reduction peaks of the PCT progressively shift towards more cathodic potentials as the medium pH increases, indicating the participation of protons in the electrochemical process [40]. As shown in Fig. 5B, a linear relationship (R² ≈ 0.99) between Eₚ and pH was acquired, providing a slope of -51 mV pH⁻¹. The proximity of this slope to the theoretical Nernst value ( ≈ − 59 mV pH⁻¹) confirms a reaction pathway involving an equal number of electrons and protons transferred, consistent with previous reports in the literature [40, 41]. It is also evident from this figure that the highest current response occurred at pH 6.

A comparative study of the supporting electrolyte was carried out by evaluating phosphate and BR buffers at equivalent concentrations and under identical experimental conditions (Figure S6). The results revealed a significantly higher current response in the presence of the BR buffer. Therefore, the BR buffer at pH 6 was chosen for the subsequent analyses, as it offered the better voltammetric performance for PCT detection.

The proposed mechanism indicates that the electrochemical oxidation of PCT involves a two-electron process, based on previously published studies [40]. A plausible reaction pathway is proposed and represented in Fig. 5C. Oxidation begins at the phenolic portion of the PCT molecule, where the loss of a proton occurs concomitantly with the transfer of an electron, forming an oxygen-centered radical delocalized on the aromatic ring. This radical intermediate subsequently undergoes intramolecular delocalization towards the acetylamino group. A second oxidation step occurs, involving the release of an additional proton and electron from the acetylamino portion. Overall, the successive two-electron and two-proton oxidation process results in the formation of N-acetyl-4-benzoquinone. As previously reported [42, 43], PCT and bismuth exhibit chemical affinity, as shown in Fig. 5D; the interaction can occur directly or indirectly with paracetamol molecules. In direct interaction, Bi^3+^ may form coordination bonds or complexes with the –OH or –C═O bonds of paracetamol or may indirectly form a stable complex structure with water. The complex may further form a hydrogen bond with the functional groups of PCT.

**Fig. 6:**
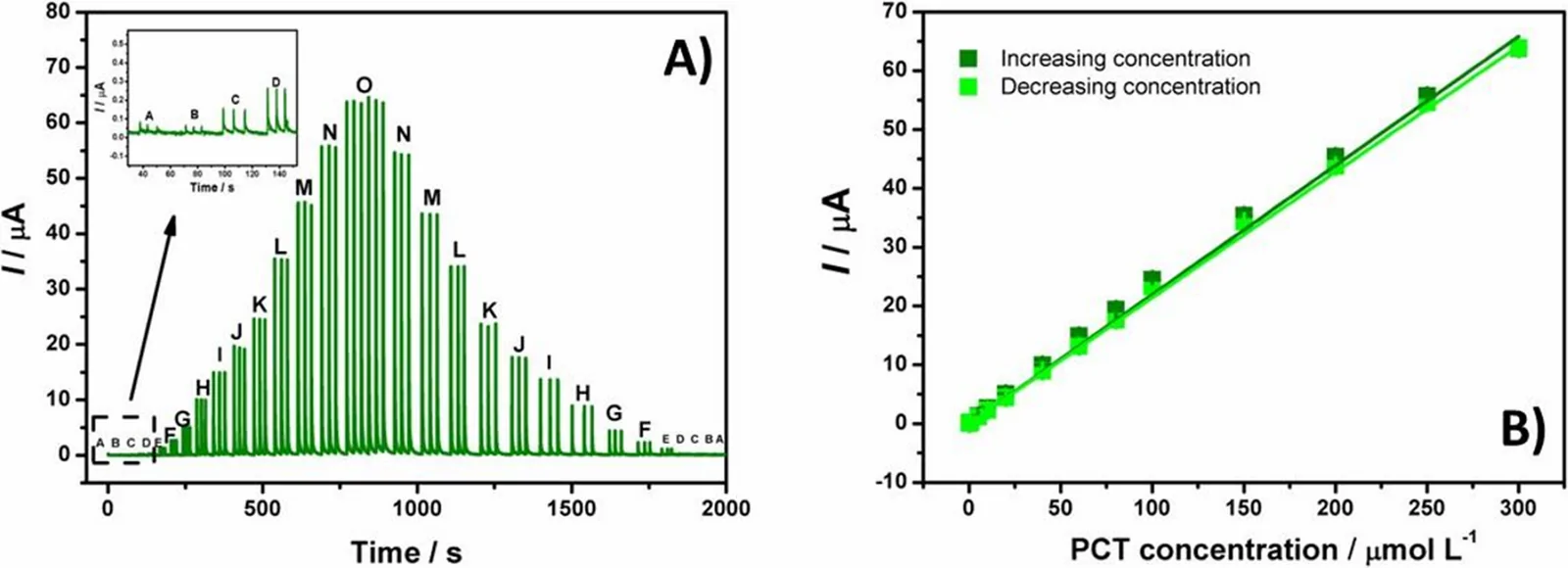
(**A**) BIA-AD recordings (*n* = 3) for increasing (A-O) and decreasing (O-A) concentrations of PCT: (A) 0.05, (B) 0.1, (C) 0.5, (D) 1.0, (E) 5.0, (F) 10.0, (G) 20.0, (H) 40.0, (I) 60.0, (J) 80.0, (K) 100.0, (L) 150.0, (M) 200.0, (N) 250.0, and (O) 300.0 µmol L^−1^ using the electrode containing 12.5% *wt.* Bi_2_O_3_NPs. (**B**) Comparison between the ascending (olive square) and descending (green square) analytical curves. BIA-AD conditions: applied potential of + 0.480 V vs. Ag|AgCl|KCl_(sat.)_, injection volume of 125 µL, and dispensing rate of 298.5 µL s^−1^ and 0.1 mol L^−1^ BR buffer (pH 6.0) as the supporting electrolyte

The mass transport properties of PCT on the G/Bi_2_O_3_NPs/PLA electrode (30:12.5:57.5% *wt.*) were investigated through a scan rate study performed in the range of 10 to 100 mV s⁻¹ (Figure S7). The dependence of the peak current on the scan rate was evaluated using the log *I* versus log *ν* plot, yielding a linear relationship. The resulting linear equation was log *I*_p_ = 0.77551 + 0.49252 log *v*, with a slope of 0.49252 ± 0.01703 and an R² = 0.99055 (Figure S7B). This value is characteristic of a diffusion-controlled mass-transport mechanism, consistent with the behavior previously reported for PCT [44, 45]. Furthermore, the Laviron equation [46] was applied to the aforementioned study, thereby determining electron-transfer parameters and confirming the participation of two electrons in the electrochemical oxidation process, with an electron-transfer coefficient (α = 0.5) [47]. These findings are fully consistent with the oxidation mechanism proposed in Fig. 6C.

### Stability of 3D printed G/Bi_2_O_3_NPs/PLA

Given the importance of durability for long-term electrochemical applications, the storage stability of the developed electrode was evaluated by comparing its current response obtained immediately after fabrication (as-printed electrode) with that recorded after 30 days of storage. During this period, the electrodes were maintained at room temperature (approximately 25 °C) and protected from both light and moisture. Prior to the post-storage measurements, the electrode surface was renewed by gentle mechanical polishing using 600- and 1200-grit sandpapers. The electrochemical response was then measured under identical conditions using a PCT solution at a concentration of 0.3 mmol L⁻¹. After 30 days of storage, a relative variation of only 2.4% in the current signal was observed compared to the initial response, demonstrating that the electrode’s electrochemical performance remains highly stable over time (Figure S8).

### Determination of PCT by the BIA technique

Given the limited availability of high-throughput electrochemical approaches for PCT determination in the literature, the BIA-AD technique is introduced as a simple, rapid, and effective alternative for analyzing pharmaceutical formulations and biological samples. Previous voltammetric tests allowed the selection of some experimental conditions for the BIA system, including the supporting electrolyte and pH. Subsequently, the effects of instrumental parameters, such as potential, injection volume, and dispensing rate, were evaluated to optimize the amperometric response for PCT determination. As shown in Figure S9, the current signal increased with the applied potential up to + 0.480 V *vs.* Ag|AgCl|KCl_(sat.)_, with no significant improvement observed at higher potentials. 125 µL injections resulted in signal stabilization. Therefore, an injection volume of 125 µL was selected for further experiments, ensuring adequate sensitivity while reducing variability and reagent consumption. Variations in the dispensing rate from 16.9 to 298.5 µL s⁻¹ altered the analytical response when a fixed injection volume was used, with the highest current signal obtained at 298.5 µL s⁻¹. Although some variability was observed, this dispensing rate provided the most efficient response and was therefore selected for subsequent amperometric measurements. The optimized parameters of the BIA-AD technique are shown in Table S4.

The linear dynamic range of the proposed method was established by injecting standard PCT solutions at increasing and decreasing concentrations into the BIA cell [48]. Figure 6A shows the amperometric responses obtained from the sequential injection of standard solutions, each analyzed in triplicate. A linear relationship was observed between the current response and the PCT concentration over the evaluated range of 0.05 to 300 µmol L^− 1^, according to the following equation: *I* = 0.07 + 0.21 C ; where (*I*) is the measured current, C is the PCT concentration, and 0.07 and 0.21 are the intercept and slope of the calibration curve, respectively), with a correlation coefficient greater than 0.999, indicating excellent linearity of the method. Additionally, based on the time required for the current signal to return to the baseline after each injection, the analytical yield was estimated at 200 injections h⁻¹. Furthermore, Fig. 6B showcases that the analytical curves obtained from the ascending and descending injection sequences exhibited very similar slope coefficients, 0.219 and 0.213, respectively. This high level of agreement between the slopes indicates the absence of a memory or fouling effect in the BIA system, confirming that previous injections do not influence the subsequent analytical response.

For comparative purposes, additional analytical curves were constructed using a non-modified electrode and an electrode modified with 12.5% *wt.* Bi_2_O_3_NPs. As shown in Figure S10, a significant increase in analytical sensitivity was achieved with the modified electrode, whose slope values were 0.058 µmol^−1^ L µA for the control electrode and 0.213 µmol^−1^ L µA for the electrode containing 12.5% *wt.* Bi_2_O_3_NPs. The limits of detection (LOD) were estimated following IUPAC recommendations [49], using the following Eq. 1:

1 *LOD=3σ/S*

in which σ is the standard deviation of the analytical response, and S is the slope of the respective calibration curve. The calculated LOD values for the unmodified and 12.5% *wt.* Bi_2_O_3_NPs-modified electrodes were 1.8 and 0.006 µmol L^−1^, respectively. Regarding the limits of quantification (LOQ) values for both electrodes, although the theoretical LOQs calculated from the calibration statistics were lower, namely 0.05 µmol L^− 1^ for the 12.5% *wt.* Bi_2_O_3_NPs-modified electrode and 5.0 µmol L^−1^ for the unmodified electrode, these values were adopted as the experimentally validated LOQs because they corresponded to the lowest concentrations included in the respective calibration ranges that provided reliable and reproducible analytical responses. The main analytical parameters obtained for the proposed method are summarized in Table 1.

**Table 1:** Analytical features achieved towards PCT sensing using the 3D-printed G/PLA (30:70% *wt.*, control electrode) and G/Bi_2_O_3_NPs/PLA (30:12.5:57.5% *wt.*)

Analytical features	3D-printed electrodes	
	G/Bi_2_O_3_NPs/PLA (30:12.5:57.5% *wt.*)	G/PLA (30:70% *wt.*)
Linear Range / µmol L^−1^	0.05–300	5–300
LOD / µmol L^−1^	0.006	1.8
LOQ / µmol L^−1^	0.05	5.0
Sensitivity / µmol^−1^ L µA	0.213	0.058
R^2^	0.999	0.995
Analytical frequency (a h ^–1^)	200	-

### Repeatability

Repeatability was assessed using 30 successive measurements at three paracetamol (PCT) concentrations (60, 80, and 250 µmol L⁻¹). The experiment was performed using injections in 10 independent replicates, as illustrated in Figure S11. The relative standard deviation (RSD) values obtained were 1.40, 2.04, and 1.25%, respectively. These results demonstrate that the electrode integrated with the BIA-AD system provides reliable and reproducible responses for PCT determination across different concentration ranges.

### Interferences

The selectivity of the method developed for real-sample analysis was evaluated through interference studies. In this context, species present in urine and water samples, as well as in pharmaceutical tablets, that could affect the electrochemical determination of PCT, such as U, GLU, GAL, SDS, and STC, were consistently investigated and selected as potential interfering species. The electrochemical response of PCT in the presence of these substances was monitored using an electrode containing 12.5% *wt.* Bi_2_O_3_NPs coupled to the BIA-AD system. The interferents were introduced individually into the electrochemical cell containing BR buffer (0.12 mol L^−1^, pH 6.0) and evaluated at interferent to PCT concentration ratios of 1:1 and 5:1. As illustrated in Figure S12, signal variations of less than 10% were observed for PCT in both ratios evaluated, demonstrating that the method exhibits satisfactory selectivity and can be reliably applied to the determination of paracetamol, even in the presence of common excipients in the samples.

### Applicability of method

**Fig. 7:**
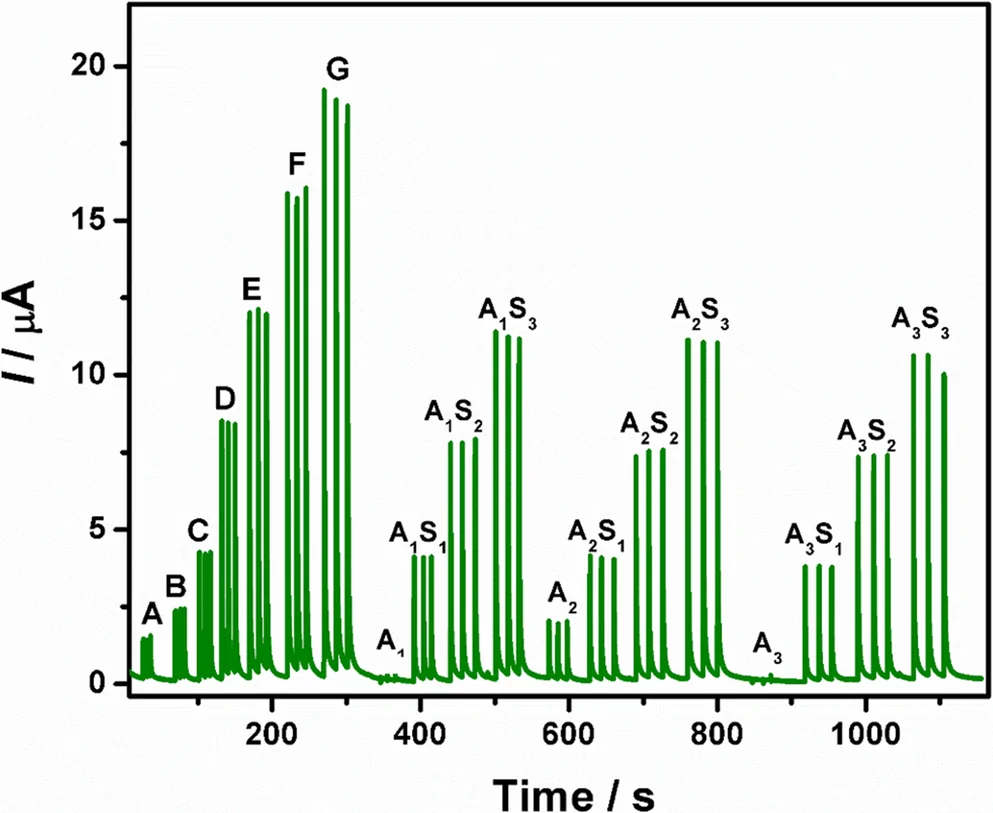
Application of the BIA-AD method for quantifying PCT in tap water (A1), pharmaceutical tablets (A2), and synthetic urine (A3) samples before and after fortification at three different PAR levels (S1, S2, and S3). The injections of A-G (5–100 µmol L^−1^) represents the obtained calibration curve for PCT. BIA-AD conditions: see Table S4

The applicability of the developed BIA method was subsequently evaluated by determining the PCT in tap water, pharmaceutical samples, and synthetic urine under optimized experimental conditions, with triplicate injections (Fig. 7). The linear working range was established by sequential injections of standard PCT solutions in increasing concentrations between 5 and 100 µmol L⁻¹ (A–G), interspersed with triplicate injections of three samples of water, pharmaceutical tablet, and synthetic urine (A1, A2, and A3, respectively), as well as samples added with three different concentration levels of standard PCT solutions (S1, S2, and S3). The proposed method identified a PCT content of 700 mg in the pharmaceutical sample, compared to the label value of 750 mg, corresponding to a recovery of 93.3%, which falls within the acceptable range for pharmaceutical analysis and confirms the method’s accuracy. In addition, recoveries for other samples (tap water and urine) ranged from 90% to 97%, indicating the accuracy of the proposed method (see Table 2). These results confirm the accuracy and reliability of the method developed to determine PCT across different samples. Furthermore, the concentrations obtained showed good agreement with those determined by the reference method (UV-Vis spectrophotometry) (Table 3). In addition, the results were statistically validated using a paired Student’s t-test at the 95% confidence level, with no statistically significant differences observed between the two methods.

**Table 2:** Results obtained for PCT determination in Tap water, pharmaceutical tablets, and synthetic urine

Samples*	Spiked / µmol L^-1^	Found / µmol L^-1^	Recovery / %
A_1_	20.0	19.16 ± 0.1	96 ± 0.9
	40.0	38.64 ± 0.1	97 ± 1.4
	60.0	57.0 ± 0.2	95 ± 1.2
A_2_	20.0	18.9 ± 0.7	94 ± 2.0
	40.0	37.3 ± 0.3	93 ± 1.1
	60.0	56.5 ± 0.3	94 ± 1.5
A_3_	20.0	18.8 ± 0.12	94 ± 1.2
	40.0	36.3 ± 0.7	91 ± 1.7
	60.0	54.2 ± 0.6	90 ± 1.6

* The samples were diluted 10-fold in the supporting electrolyte.

*Manufacturer's label value: 750 mg / tablet

A_1_: Tap water

A_2_: Pharmaceutical tablet

A_3_: Synthetic urine

**Table 3:** PCT contents in tap water, pharmaceutical tablet, and synthetic urine were determined by BIA-AD and UV–vis methods

Sample*	BIA-AD	UV-vis
A_1_	19.16 ± 0.1	21.13 ± 0.01
	38.64 ± 0.1	42.20 ± 0.01
	57.0 ± 0.2	60.57 ± 0.01
A_2_	18.9 ± 0.7	19.43 ± 0.02
	37.3 ± 0.3	38.33 ± 0.02
	56.5 ± 0.3	58.33 ± 0.02
A_3_	18.8 ± 0.12	18.48 ± 0.01
	36.3 ± 0.7	39.35 ± 0.01
	54.2 ± 0.6	56.63 ± 0.01

*Student's test: t_cal_ =3.55 < t_crit_ =4.30 at a 95 % confidence level.

A_1_: Tap water

A_2_: Pharmaceutical tablet

A_3_: Synthetic urine

The analytical performance of the proposed electrode containing 12.5% *wt.* Bi_2_O_3_NPs were critically benchmarked against electrochemical sensors previously reported for PCT determination, considering key parameters such as sensor configuration, electroanalytical technique, linear working range, and limits of detection (LODs), as summarized in Table 4. The developed platform exhibited a wide linear dynamic range (0.05–300 µmol L⁻¹), which is broader than those reported for most conventional modified electrodes, including graphene/GCE [50], 3DAuPd/GN-CNTs-IL [51], and HA-Fe₃O₄/SPCE [52] systems. This extended working range is particularly advantageous for applications involving samples with variable or elevated PCT concentrations, reducing the need for dilution steps and expanding the sensor’s practical applicability. In terms of detectability, the proposed sensor achieved a low LOD of 0.006 µmol L⁻¹, which is comparable to or even slightly superior to those obtained using more complex nanostructured platforms, such as HA-Fe₃O₄/SPCE (0.0065 µmol L⁻¹) and significantly lower than values reported for PEDOT/AG/GCE [53], Cu-MOFs/graphene/GCE [38], PPy/PPa-PGE [54] and ACeO_2_@GNR/IJPCNT [55]. These results indicate that incorporating Bi_2_O_3_NPs into the G/PLA matrix provides an efficient interface for PCT oxidation, thereby enhancing signal intensity and lowering the LOD without the need for noble metals or intricate multilayer assemblies. Moreover, while several sensors reported in the literature rely on sophisticated fabrication routes, noble metal nanoparticles, or enzyme-based architectures, the present platform combines additive manufacturing with low-cost conductive composites and a straightforward surface-modification strategy. The use of BIA-AD further enhances analytical performance by enabling rapid measurements, high sample throughput, and reduced reagent consumption, which are attractive features for routine and decentralized analysis. Overall, the proposed 3D-printed G/Bi_2_O_3_NPs/PLA electrode stands out as a competitive alternative to conventional electrochemical sensing platforms for PCT determination, offering a favorable balance between analytical performance, operational simplicity, cost-effectiveness, and scalability. These characteristics position the developed sensor as a promising tool for practical applications in PCT monitoring across clinical, pharmaceutical, and environmental settings.

**Table 4:** An overview of the analytical performance of the proposed sensor toward PCT detection, compared with electrochemical systems previously described in the literature

Sensor	Method	Linear range / µmol L^−1^	LOD / µmol L^−1^	Ref.
Cu-MOFs/graphene/GCE	DPV	0.2–160	0.016	[56]
ACeO2@GNR/IJPCNT	SWV	1–100	0.18	[55]
PEDOT/AG/GCE	Amperometry	0.15–5880	0.041	[53]
PPy/PPa-PGE	DPV	15–150	3.45	[54]
3D AuPd/GN-CNTs-IL	DPV	0.1–50.0	0.050	[51]
Graphene/GCE	SWV	0.1–20	0.032	[50]
HA-Fe_3_O_4_/SPCE	DPV	0.025-20	0.0065	[52]
3D-printed G/Bi_2_O_3_NPs/PLA electrode	BIA-AD	0.05–300	0.006	This work

Cu-MOFs: copper-metal-organic frameworks; ACeO_2_@GNR/IJPCNT: amidase/cerium dioxide@graphene nanoribbon composite modified disposable inkjet-printed carbon nanotubes electrode; PEDOT/AG: gold@graphene core-shell nanoparticles doped poly(3,4-ethylenedioxythiophene); PPy/PPa: polypyrrole/polypyrrole-3-carboxylic acid copolymer; 3D AuPd/GN-CNTs-IL: three-dimension gold palladium nanoparticles-ionic liquid functionalized graphene-carbon nanotubes; Graphene/GCE: graphene-modified glassy carbon electrode; HA-Fe_3_O_4_/SPCE: humic acid functionalized iron oxide nanoparticles SWV: square wave voltammetry; DPV: differential pulse voltammetry; BIA-AD: batch injection analysis with amperometric detection

### Electrochemical determination of lead

**Fig. 8:**
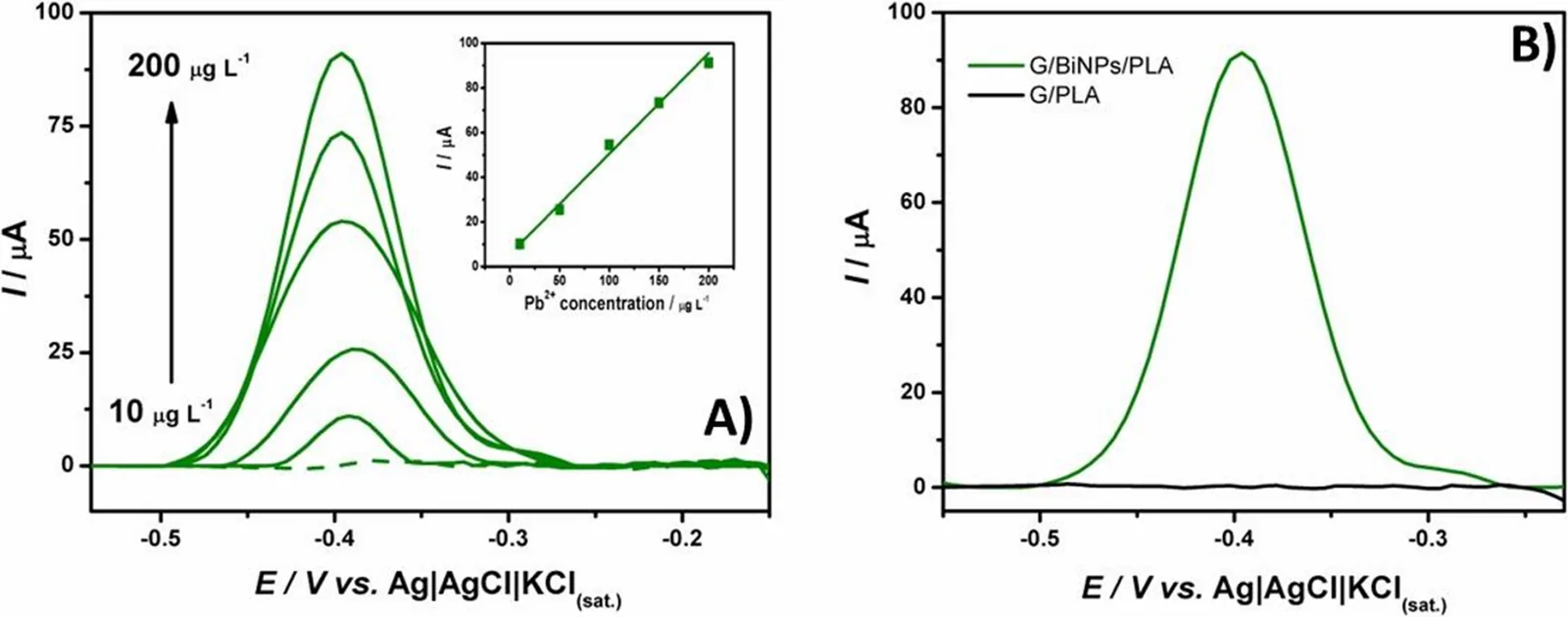
(**A**) Baseline-corrected SWV recordings (*n* = 3) at increasing Pb(II) concentrations (10 to 200 µg L^−1^) on the Bi_2_O_3_NPs-modified 3D-printed sensor (12.5% *wt.*). (**B**) Baseline-corrected SWV recordings (*n* = 3) using 200 µg L^−1^ of Pb(II) on the Bi_2_O_3_NPs-modified 3D-printed (12.5% *wt.*, olive line) and G/PLA (30:70% *wt.*, black line) electrodes

Bi-based electrodes have demonstrated outstanding performance at anodic potentials, allowing highly sensitive stripping voltammetric detection of trace-level metals [7, 57]. Therefore, the analytical performance of the Bi_2_O_3_NPs-modified 3D-printed sensor (12.5% *wt.*)and G/PLA (30:70% *wt.*)was tested for the determination of Pb(II) by square-wave voltammetry (SWV) as a proof of concept. The analytical curves for Pb(II) using SWV were obtained over the concentration range of 10 to 200 µg L^−1^ for both electrodes in 0.01 mol L^−1^ HCl (pH 2.0), with a pre-concentration step at − 1.2 V for 180 s. The corresponding voltammograms and calibration curve are shown in Fig. 8. A coefficient of determination (R²) of 0.992 was obtained over the evaluated concentration range for the Bi_2_O_3_NPs-modified electrode, according to the following equation: *I* = 5.5 + 0.44 C; where (*I*) is the measured current, C is the Pb(II) concentration, and 0.55 and 0.44 are the intercept and slope of the calibration curve, respectively. The LOD value was estimated according to Eq. 4 following IUPAC recommendations [49]. The LOQ value was also calculated following the IUPAC recommendations [49], using Eq. 2:

2 *LOQ=10σ/S*

where σ is the standard deviation of the analytical response, and S is the slope of the respective calibration curve. The estimated LOD and LOQ values for the Bi_2_O_3_NPs-modified electrode were 1.81 µg L^−1^ and 5.4 µg L^−1^. It is also possible to highlight that the non-modified electrode (G/PLA (30:70% *wt.*)) was not able to detect concentrations in the range of 10 to 200 µg L^−1^, whereas the electrode containing 12.5% *wt.* Bi_2_O_3_NPs exhibited a clear and measurable response within this concentration range, as shown in Fig. 8B.

The repeatability of the method was evaluated through seven consecutive DPV measurements (*n* = 7) using Pb(II) solutions at concentrations of 10 and 150 ppb with the G/Bi_2_O_3_NPs/PLA electrode containing 12.5% *wt.* Bi_2_O_3_NPs. The results demonstrated high consistency among measurements, highlighting the good stability of the electrochemical response. The relative standard deviation (RSD) values obtained for the 10 and 150 ppb concentrations were 7.8% and 8.1%, respectively. Reproducibility across different electrodes was determined using three independent G/Bi_2_O_3_NPs/PLA electrodes prepared under the same experimental conditions. An RSD value of 3.2% was obtained, indicating adequate agreement between analytical responses and demonstrating the reproducibility of the surface across different Bi_2_O_3_NPs sensors. A selectivity study was conducted to evaluate the effect of potentially interfering ions on Pb(II) determination using the Bi_2_O_3_NPs-based electrode. To this end, Cu²⁺, Zn²⁺, and Cd²⁺ were evaluated individually at a 1:1 ratio relative to the analyte. As shown in Fig. S13A–C, the voltammograms obtained in the presence of the interferents showed no significant changes in the Pb(II) peak currents. Furthermore, Fig. S13D demonstrates that the relative Pb(II) responses in the absence and presence of the interferents remained statistically similar, confirming that Cu²⁺, Zn²⁺, and Cd²⁺ did not cause significant interference in the determination of Pb(II).

A real tap water sample was analyzed to evaluate the applicability of the proposed method for Pb(II) determination. All measurements were performed in triplicate (*n* = 3) using the standard addition method to minimize potential matrix effects. Since the Pb(II) concentration in the sample was below the method’s detection limit, recovery experiments were performed by spiking the sample with Pb(II) at 10 µg L⁻¹. The SWV voltammograms recorded during the standard addition procedure and the corresponding calibration plots obtained for Pb(II) determination are presented in Fig. S13A and Fig. S13B, respectively. A recovery value of 104 ± 2% was achieved, demonstrating the excellent accuracy and reliability of the proposed method for Pb(II) determination in real water samples. This result further highlights the robustness, versatility, and practical applicability of the developed sensor, reinforcing its potential for environmental monitoring and for determining trace levels of lead in complex aqueous matrices.

It is important to highlight that the determination of PCT and Pb(II) was not intended to represent two independent studies, but rather to demonstrate the multifunctionality of the same G/Bi₂O₃NPs/PLA platform. The rationale is based on the well-established versatility of bismuth-based materials in electroanalysis. In the case of PCT, incorporating Bi₂O₃ nanoparticles improves interfacial electron-transfer properties and promotes the electrochemical oxidation of the organic molecule. In contrast, the same bismuth-containing surface provides favorable characteristics for the preconcentration and stripping determination of Pb(II). Thus, the two applications were included to demonstrate that the proposed filament can support electrochemical sensing of chemically distinct analytes using different electroanalytical mechanisms. In this way, the Pb(II) experiment was intentionally presented as a proof-of-concept application rather than as a second fully optimized analytical method.

## Conclusion

This study presents a multifunctional G/Bi_2_O_3_NPs/PLA composite designed for fabricating 3D-printed electrochemical sensing platforms. The filament was obtained via a simple, rapid route in which a Bi precursor was converted into bismuth Bi_2_O_3_NPs directly within the polymeric matrix. A combination of spectroscopic, morphological, elemental, and electrochemical analyses confirmed the successful formation of a conductive hybrid material and highlighted the effective integration of G and Bi domains throughout the PLA structure. The electrochemical behavior of the fabricated sensors demonstrated that the synergistic interaction between the carbonaceous framework and the dispersed Bi_2_O_3_NPs markedly enhances charge-transfer kinetics and mass transport, thereby improving analytical performance for PCT detection.

Exploiting these features, a practical and sensitive amperometric method was developed and successfully applied to the analysis of real samples. The proposed method exhibited reliable analytical figures of merit, including high precision (RSD < 5%), suitable accuracy (recoveries between 90 and 97%), and robust selectivity in the presence of possible interferents. In addition, the long-term stability of the G/Bi_2_O_3_NPs/PLA electrode was systematically evaluated over 30 days. The sensor retained its electrochemical response with minimal signal fluctuations, showing only a 2.44% variation in current, demonstrating the excellent stability and durability of the proposed sensing platform.

Furthermore, the applicability of the developed platform was also extended to Pb(II) detection, demonstrating the versatility of the G/Bi_2_O_3_NPs/PLA composite and revealing an additional potential application for environmental monitoring and heavy metal analysis. Beyond its analytical performance, the proposed strategy highlights the potential of in situ metal nanoparticle formation within thermoplastic filaments as a sustainable route for the additive manufacturing of electrochemical devices. The developed Bi_2_O_3_NPs-based composite filament thus constitutes a flexible and scalable platform for next-generation electrochemical sensors, with promising applicability in pharmaceutical quality control, tap water analysis, and biological sample monitoring.

## Supplementary Information

Below is the link to the electronic supplementary material.

Supplementary Material 1

## Data Availability

No datasets were generated or analysed during the current study.

## Declarations

### AI Usage Statement

The authors used artificial intelligence (AI)-assisted tools exclusively to improve grammar, language clarity, and text readability. All scientific content, data analysis, interpretation of results, conclusions, and final manuscript revisions were performed by the authors. The authors take full responsibility for the accuracy, integrity, and originality of the work.
