# Supplementary material for: An advanced carbon/bismuth oxide nanoparticle-based filament for multifunctional 3D-printed electrochemical sensors toward paracetamol and lead in pharmaceutical, clinical, and environmental electroanalysis
Source: Mikrochim Acta. 2026 Sep 14;193(10):681. doi: 10.1007/s00604-026-08401-z (PMC13574912; doi:10.1007/s00604-026-08401-z)
Supplement: Supplementary file 1 [file 604_2026_8401_MOESM1_ESM.docx]

**An advanced carbon/bismuth oxide nanoparticle~~s~~-based filament for multifunctional 3D-printed electrochemical sensors toward paracetamol and lead in pharmaceutical, clinical, and environmental electroanalysis**

Natália Marinho Caldas ^a^, Amanda Garcia Batista ^a^, Daniel M.-D. L. Alves^a^, Samuel C. Silva^b^, Diego A. Peixoto^b^, Ariadne C. Catto^c^, Valmor R. Mastelaro^c^ Edson Nossol^b^, Lucas V. de Faria^d^, Diego P. Rocha^e^, Bruno Campos Janegitz ^f^, Rafael Machado Dornellas^a^ *

^a^ Department of Analytical Chemistry, Institute of Chemistry, Fluminense Federal University, Niterói, 24020-141, RJ, Brazil.

^b^ Institute of Chemistry, Uberlândia Federal University, Uberlândia, 38408-100, MG, Brazil.

^c^ Institute of Physics of São Carlos, University of São Paulo, 13566-590, São Carlos, SP, Brazil.

^d^ Department of Analytical Chemistry, Institute of Chemistry, Federal University of Rio de Janeiro, Rio de Janeiro 21941-909, Brazil.

^e^ Department of Chemistry, Federal Institute of Paraná, Pitanga 85201-106, Paraná, Brazil.

^f^ Department of Chemistry, Federal University of São Carlos, São Carlos 13565-905, SP, Brazil.

*Corresponding author

Email address: [rafaeldornellas@id.uff.br](mailto:rafaeldornellas@id.uff.br)

**Fig. S1 -** EDS spectra of (a) material and the electrodes containing PLA/G and Bi ((b) 7.5, (c) 10, and (d) 12.5 wt.%).

**Fig. S2**- (A) High-resolution C 1s spectrum and (B) High-resolution O 1s spectrum.

**Fig. S3** (A) Electrochemical behavior of G/PLA (black line), G/Bi_2_O_3_NPs/PLA (30:7.5:62.5% *wt.*, red line), G/Bi_2_O_3_NPs/PLA (30:10:60 % *wt.*, blue line), G/Bi_2_O_3_NPs/PLA (30:12.5:57.5 % *wt.*, olive line) electrodes in 0.1 mol L^-1^ KCl (supporting electrolyte); (B) Electrochemical response of five (n = 3) different 3D printed G/Bi_2_O_3_NPs/PLA (30:12.5:57.5 % *wt.*) electrodes in the presence of the same supporting electrolyte. Experimental conditions: scan rate of 50 mV s^-1^; step of potential 5 mV.

**Fig. S4**  (A) Cyclic voltammograms obtained for 1 mmol L^-1^ [Fe(CN)_6_]^4-/3-^ in 0.1 mol L^-1^ KCl (background electrolyte) upon the (A) G/PLA (30:70% *wt.*, black line), (B) G/Bi_2_O_3_NPs/PLA (30:7.5:62.5 % *wt.*, red line), (C) G/Bi_2_O_3_NPs/PLA 30:10:60 % *wt.*, blue line) and (D) G/Bi_2_O_3_NPs/PLA (30:12.5:57.5 % *wt.*, olive line) electrodes.(B) EIS spectra acquired for (a) G/PLA (black square), G/Bi_2_O_3_NPs/PLA (30:7.5:62.5 % *wt.*, red square), G/BiNPs/PLA (30:10:60 % *wt.*, blue line), G/Bi_2_O_3_NPs/PLA (30:12.5:57.5 % *wt.*, olive line)) electrode surfaces using 1 mmol *L*^−1^ redox probe [Fe(CN)_6_]^4-/3-^ in 0.1 mol *L*^−1^ KCl (supporting electrolyte). The inset corresponds to the magnification of the spectra using G/PLA and G/Bi_2_O_3_NPs/PLA electrodes and the associated Randles equivalent circuit. EIS conditions: frequency between 50,000 Hz and 0.01 Hz, 10 data points per decade, and amplitude of 10 mV. Experimental conditions: recorded at a scan rate of 50 mV s⁻**¹** within the potential range of −0.2 to 0.7 V and a step potential of 5 mV.

**Fig. S5** Cyclic voltammograms obtained for 1 mmol L^-1^ [Fe(CN)_6_]^4-/3-^ in 0.1 mol L^-1^ KCl (background electrolyte) upon the (A) G/PLA (30:70 % *wt.*, black line), (B) G/Bi_2_O_3_NPs/PLA (30:7.5:62.5 % *wt.*, red line), (C) G/Bi_2_O_3_NPs/PLA 30:10:60 % *wt.*, blue line) and (D) G/Bi_2_O_3_NPs/PLA (30:12.5:57.5 % *wt.*, olive line) electrodes at scan rates in the range of 10 to 100 mV s^-1^; the insets show the respective linear fits between peak current and the square root of the scan rate. Experimental conditions: The potential range of −0.2 to 0.7 V and a step potential of 5mV.

**Fig. S6** CV recordings of the 0.3 mmol L-1 PCT electrode upon G/Bi_2_O_3_NPs/PLA (30:12.5:57.5 % *wt.*) using 0.12 mol L^-1^ phosphate buffer (pH = 6.0, purple line) and 0.12 mol L-1 BR buffer (pH = 6.0, olive line) as supporting electrolytes. The dashed lines correspond to the respective blanks. Experimental conditions: recorded at a scan rate of 50 mV s⁻**¹** within the potential range of −0.3 to 1.0 V and a step potential of 5mV.

**Fig.** S7 (A) CVs obtained for 0.3 mmol L^-1^ PCT in 0.12 mol L^-1^ BR buffer (pH 6.0) utilizing G/Bi_2_O_3_NPs/PLA (30:12.5:57.5 % *wt.*) electrode with scan rates ranging from 10 to 100 mV s^-1^; (B) Linear adjustment between Log I vs. Log ν.; (C) *E_p_* *vs.* log *v*. Background electrolyte: 0.12 mol L^-1^ BR buffer (pH 6.0). Experimental conditions: recorded at a scan rate of 50 mV s⁻**¹** within the potential range of −0.1 to 1.0 V and a step potential of 5mV.

**Fig S8. (A)** Electrochemical responses recorded for 0.3 mmol L^-1^ PCT in 0.12 mol L^-1^ BR buffer (pH = 6.0 (background electrolyte) using the 3D-printed electrodes on the first day (magenta line), after a 15-day storage period (olive line), and after a 30-day storage period (purple line). The dashed lines illustrate the corresponding background electrolyte: 0.12 mol L^-1^ BR buffer (pH 2.0). Experimental conditions: recorded at a scan rate of 50 mV s⁻**¹** within the potential range of −0.1 to 1.0 V and a step potential of 5mV. (B) Stability of the electrochemical response, expressed as the current measured on Day 1, Day 15, and Day 30.

**Fig S9.** Impact of BIA-AD parameters: (A) applied potential (+0.40 to +0.51), (B) injection volume (25 to 200 µL), and (C) dispensing rate (16.9 to 298.5 µL s^-1^) on the electrochemical response of 30.0 μmol L^-1^ PCT in 0.12 mol L^–1^ BR buffer (pH 6) using the G/Bi_2_O_3_NPs/PLA electrode (30:12.5:57.5 % *wt.*).

**Fig. S10.** Analytical curves obtained for PCT between 0.05 and 300 µmol L^-1^ using the G/PLA (30:70 % *wt.*, black line) and G/Bi_2_O_3_NPs/PLA (30:12.5:57.5 % *wt.*, Olive line) electrodes by the BIA-AD technique. BIA-AD parameters: see Table S4.

**Fig. S11** BIA-AD records obtained for quintuplicate injections of PCT at three concentration levels (a) 60 µmol L^-1^, (b) 80 µmol L^-1^, and (c) 250 µmol L^-1^ employing the G/Bi_2_O_3_NPs/PLA (30:12.5:57.5 % *wt.*) electrode. BIA-AD parameters: see Table S4.

**Fig. S12** BIA-AD responses recorded for 60 µmol L^-1^ PCT in 0.12 mol L^-1^ BR buffer (pH 6.0) solution (supporting electrolyte) in the absence and presence of interferents (GLU, AMD, SDS, GAL, and U) using (A) 1:1 and (B) 5:1 ratios between PCT and interfering compounds. The influence on the PCT relative responses is shown in (C) and (D). BIA-AD parameters: see Table S4.

**Fig. S13** SWV responses recorded for 150 µmol L^-1^ Pb (II) in 0.01 mol L^-1^ HCl (pH 2.0) in the absence and presence of interferents (Cd²⁺, Zn²⁺ and Cu²⁺) using 1:1 ratios between Pb^2+^ and interfering compounds. The influence on the Pb²⁺ relative responses is shown in (D).

**Fig. S14** SWV responses (n = 3) obtained for tap water sample (A) using a 3D-printed G/Bi_2_O_3_NPs/PLA electrode (30:12.5:57.5 % *wt.*) and the respective calibration plots by the standard addition method (B). The voltammograms show the non-spiked sample (green dashed lines), and the spiked sample at 10.0 µmol L−1 Pb (II) (green line), followed by the three standard additions**.**

**Table S1 –** I_D_/I_G_ ratio of PLA/G material and electrodes with varying Bi content.

| **Electrodes** | I_D_/I_G_ |
| --- | --- |
| G/PLA (30:70 % *wt.*) | 0.48 |
| G/Bi_2_O_3_NPs/PLA (30:7.5:62.5 % *wt.*) | 0.61 |
| G/Bi_2_O_3_NPs/PLA (30:10:60 % *wt.*) | 0.39 |
| G/Bi_2_O_3_NPs/PLA (30:12.5:57.5 % *wt.*) | 0.43 |

**Table S2.** Electrochemical data acquired for the 1 mmol L^-1^ [Fe(CN)_6_]^4-/3-^ redox probe using the proposed working electrodes.

| **Electrodes** | ***I*_pc_ / µA** | ***I*_pa_ / µA** | **Δ*E_p_* / V** | **A_elet_ / cm^2^** | **K^0^** |
| --- | --- | --- | --- | --- | --- |
| G/PLA (30:70 % *wt.*) | -8.16 | 11.01 | 0.385 | 1.34 | 1.80x10^-4^ |
| G/Bi_2_O_3_NPs/PLA (30:7.5:62.5 % *wt.*) | -12.78 | 14.45 | 0.145 | 1.71 | 1.36x10^-3^ |
| G/Bi_2_O_3_NPs/PLA (30:10:60 % *wt.*) | -20.78 | 21.94 | 0.125 | 3.48 | 1.69x10^-3^ |
| G/Bi_2_O_3_NPs/PLA (30:12.5:57.5 % *wt.*) | -23.41 | 25.64 | 0.095 | 4.82 | 2.19x10^-3^ |

**Table S3 -** Anodic and cathodic peaks of PCT current values achieved from Figure 4 using the proposed electrodes.

| **Electrode** | ***I*_p_ (**µA**)** | | ***I_c_* (**µA**)** | **Δ*E_p_* / V** |
| --- | --- | --- | --- | --- |
| G/PLA (30:70 % *wt.*) |  | 8.09 | -2.86 | 0.470 |
| G/Bi_2_O_3_NPs/PLA (30:7.5:62.5 % *wt.*) |  | 11.82 | -7.86 | 0.110 |
| G/ Bi_2_O_3_NPs/ PLA (30:10:60 % *wt.*) |  | 30.64 | -18.49 | 0.100 |
| G/Bi_2_O_3_NPs/PLA (30:12.5:57.5 % *wt.*) |  | 39.95 | -24.11 | 0.085 |

**Table S4.** Range studied and values selected for sensing of PCT by the BIA-AD system using the 3D printed G/Bi_2_O_3_NPs/PLA (30:12.5:57.5 % *wt.*) electrode.

| **Parameters** | **Studied range** | **Selected value** |
| --- | --- | --- |
| Applied potential *vs.* Ag\|AgCl\|KCl_(sat.)_ / V | +0.40 to +0.51 | + 0.480 |
| Dispensing rate / µL s^-1^ | 16.9 – 298.5 | 298.5 |
| Volume / µL | 25 – 200 | 125 |
